# Landing Biomechanics in Patients 2 Years After Augmented ACL Repair and 2 Years After Hamstring Autograft ACL Reconstruction Compared With Controls

**DOI:** 10.1177/23259671251358386

**Published:** 2025-07-25

**Authors:** Linda Bühl, Sebastian Müller, Corina Nüesch, Florian Samuel Halbeisen, Annegret Mündermann, Christian Egloff

**Affiliations:** † Department of Biomedical Engineering, University of Basel, Basel, Switzerland; ‡ Neuroorthopaedics and Centre for Clinical Motion Analysis, University Children's Hospital Basel (UKBB), Basel, Switzerland; § Department of Orthopaedics and Traumatology, University Hospital Basel, Basel, Switzerland; ‖ Altius Swiss Sportmed Center AG, Rheinfelden, Switzerland; ¶ Department of Spine Surgery, University Hospital Basel, Basel, Switzerland; # Department of Clinical Research, University of Basel, Basel, Switzerland; ** Surgical Outcome Research Center, University Hospital and University Basel, Basel, Switzerland; †† Department of Teaching, Research and Development, Schulthess Clinic, Zurich, Switzerland; ‡‡ Department of Hip and Knee Surgery, Schulthess Clinic, Zurich, Switzerland; Investigation performed at the Department of Orthopaedics and Traumatology at the University Hospital Basel, Basel, Switzerland

**Keywords:** anterior cruciate ligament, functional assessment, InternalBrace, kinematics and kinetics, side-to-side difference, single-leg hop

## Abstract

**Background:**

InternalBrace-augmented anterior cruciate ligament repair (ACL-IB) is believed to restore natural knee mechanics. However, there is a dearth of data on in vivo leg biomechanics after ACL-IB and comparability with gold standard surgery.

**Purposes:**

To (1) investigate differences in sagittal and frontal landing biomechanics of the legs in patients after ACL-IB (Comparison I) and after anterior cruciate ligament reconstruction (ACLR; Comparison II), compare the involved legs with controls (Comparison III), and (2) identify leg differences that were greater than those typically observed in controls.

**Study Design:**

Cross-sectional study, Level of Evidence 3.

**Methods:**

A total of 29 patients who had ACL-IB, 27 sex- and age-matched patients who had ACLR, and 29 matched controls were asked to perform single-leg hops (SLH) for maximum forward distance 2 years postoperatively, assessed by marker-based motion analysis. Sagittal (hip, knee, and ankle) and frontal (hip and knee) plane angles and peak moments, joint work contribution, and peak vertical ground-reaction force and loading rates during landing were calculated. Differences between the involved and uninvolved legs in patients (paired *t* tests) and between the involved legs in patients and the nondominant legs in controls (1-way analysis of variance) were analyzed. To determine whether these differences exceeded the typical variation seen in the control legs, we compared the overlap of the 95% CIs of the differences with the 95% CIs of the within-control differences (the nondominant versus dominant leg).

**Results:**

Patients who underwent ACL-IB (ACL-IB group) and ACLR (ACLR group) showed significant differences in their legs’ SLH landing biomechanics. Only leg differences in the ACL-IB group were greater than those in in the control group (no overlap): smaller peak knee flexion angle (leg difference: −8.3° [95% CI, −13 to −3.7]; *d* = −0.85; *P* = .001; controls [95% CI, −1.7 to 4.2]) and lower peak knee flexion moment (−0.60 Nm/kg [95% CI, −0.72 to −0.31]; *d* = −0.72; *P* < .001; controls [95% CI, −0.06 to 0.35]) in the involved compared with the uninvolved leg; and lower peak knee flexion moment in the involved leg compared with control legs (−0.50 Nm/kg [95% CI, −1 to −0.07]; *d* = −0.71; *P* = .020).

**Conclusion:**

Persistent differences in SLH landing biomechanics 2 years after ACL surgery suggest that ACL ruptures cause alterations that cannot be restored by augmented repair or reconstruction alone. The greater differences between the legs in the ACL-IB group than those typically seen within controls highlight the need for further research to understand the full potential or limitation of ACL preservation techniques.

In recent years, there has been increasing interest in alternative treatments that may improve outcomes after anterior cruciate ligament (ACL) surgery.^17,37^ As a result, augmented ACL repair for proximal ACL tears has emerged, in which the torn ligament is reattached to the femur and synthetically reinforced with a tape augmentation (InternalBrace; Arthrex Inc).^59^ Unlike the gold standard of ACL reconstruction (ACLR), this procedure preserves the native ACL fibers and does not require tendon harvesting.^59^ A limited number of retrospective or case-control studies have shown comparable patient-reported and clinical outcomes (eg, passive anterior knee stability) between ACL-IB and ACLR.^12,14,16,23,35,41,48,56,60,61^ Only a few of those studies report on functional outcomes such as thigh muscle strength, hop or balance performance between patients who had ACL-IB and ACLR, supporting comparable results for these parameters.^14,16,35^ Because of the preservation of the native ACL fibers and promising results in cadaveric knee kinematics after ACL-IB compared with ACLR,^9,53^ proponents of ACL repair believe that natural knee mechanics are preserved.^51,59^ However, human in vivo studies on knee biomechanics after ACL-IB, and, to the best of our knowledge, comparisons of leg biomechanics after ACL-IB with the gold standard ACLR are lacking.

During ACL injury rehabilitation, specific functional tests, depending on the physical demands required, are used to determine when it is appropriate for an individual to return to activities, sports, or competition,^64^ and therefore play a key role in assessing these milestones in the recovery process. Some of the most commonly used functional tests after ACLR are single-leg hop (SLH) tests.^3,24^ In addition to SLH performance (the quantitative outcome of the task; eg, SLH distance),^3^ joint biomechanics (the behavior and quality of movement execution related to how this performance is achieved; eg, peak knee flexion or power generation/absorption) have been studied after ACLR.^24,27^ After ACLR, biomechanical alterations persist in the involved compared with the uninvolved knee and/or the knees of controls^24,27,36^ and may induce changes in the ipsilateral leg joints^15,40^ or the contralateral leg.^28,36^ Despite “normal” or comparable performance to the uninjured leg after ACLR (ie, symmetrical SLH distances), evidence suggests nonsymmetrical biomechanics^27,63,66^ and a persistent risk of recurrent ACL injury.^38,62^ Therefore, biomechanical analyses and the way movement is performed and performance is achieved may provide a deeper insight into possible compensatory or shifting mechanisms that predispose patients to injury.

While performance in hop distance in the involved leg can be compared with the uninvolved leg after ACL surgery, it has been questioned to what extent the contralateral leg is affected by unilateral ACLR and whether this leg can be a suitable reference.^19,50^ Moreover, minimal clinically important differences based on healthy controls or reliability measures for biomechanical parameters are often reported for walking than for other dynamic movement tasks.^11,42^ Therefore, a healthy reference group is important to identify relevant changes after ACL injury or surgery, especially during dynamic, athletic activities where ACL injuries are common, such as single-leg landings.^4^ Using the concept of noninferiority or equivalence studies, a confidence interval or its limits, for example, from healthy individuals, can be used as a reference range or a cutoff value for at most irrelevant deviations.^13,31,52^ Hence, it can be assumed that values within a 95% CI of leg differences in healthy controls (no previous surgery or reported symptoms in their lower legs) have no relevant meaning and that this range can be considered as a typical, natural variation in leg symmetry. Accordingly, leg differences in patients falling within this range would also reflect at most an irrelevant asymmetry, whereas leg differences in patients falling outside this reference range could most likely be considered as atypical changes^45^ with potential (clinical) relevance.^13^

Overall, information on tibiofemoral motion and relevant differences in the legs of patients compared with controls is needed to demonstrate the full potential and/or limitations of the emerging ACL repair techniques compared with the gold standard ACLR.

Therefore, the primary aim of this study was to investigate differences in sagittal plane hip, knee, and ankle kinematics and kinetics, frontal plane hip and knee kinematics and kinetics, and vertical ground-reaction force (GRF) during SLH landing for the following comparisons:

- Comparison between the involved and uninvolved leg in the ACL-IB group (within-patient difference in ACL-IB)
- Comparison between the involved and uninvolved leg in the ACLR group (within-patient difference in ACLR)
- Comparison between the involved leg in the ACL-IB group, the involved leg in the ACLR group, and the nondominant leg in the control group (between-group differences).

Based on the literature,^24,27,36^ we expected within-patient differences in kinematics and kinetics after ACLR and between-group differences after ACLR compared with the control group. Comparable clinical and patient-reported outcomes and hop distances have been reported after ACL-IB and ACLR, with lower values compared with the controls.^7,16,35,44^ Therefore, we also expected within-patient differences in the ACL-IB group, and between-group differences between the ACL-IB and control groups, but not between the ACL-IB and ACLR groups.

In addition, our secondary aim was to determine whether the magnitude of biomechanical deviations observed within patients and between groups was greater than the typical variations in controls and therefore potentially clinically relevant.

## Methods

This study is part of a larger nonrandomized case-control umbrella project (RetroBRACE II),^43^ approved by the Ethics Committee of Northwestern and Central Switzerland (EKNZ 2019-00491 and 2020-00551), and registered at clinicaltrials.gov (NCT04429165).

### Participants

Hospital cases at our institution between May 2016 and April 2020 were screened for eligible patients with proximal rupture 2 years after primary unilateral ACL-IB (Sherman classification type I-II^54^) or patients 2 years after ACLR using 4-folded hamstring tendons. Patients after ACL-IB had to undergo surgery within a maximum of 5 weeks after the index injury. Patients after ACLR had to undergo surgery within a maximum of 8 months after the index injury. These patients were matched for sex and age (with a maximum age difference of ±4 years). The exclusion criteria for all patients were concomitant rupture of the posterior cruciate ligament or complete rupture of both collateral ligaments, previous injury/surgery of the involved leg within the previous 6 months, or previous surgery of the uninvolved leg. Sex- and age-matched healthy controls were recruited in the surrounding area. The inclusion criteria for controls were no previous lower limb or knee ligament injury.^43^ Further details on the recruitment strategy have been described in the Müller et al study.^44^ All participants provided written informed consent.

### Study Protocol

Participants were tested at the Laboratory of Functional Biomechanics at the University Hospital Basel (single visit). Participants performed the test for maximum forward SLH distance, with each leg in their shoes in a randomized order on a walkway with 2 force plates embedded next to each other (Kistler force plate 9260AA6, Kistler AG, Winterthur, CH; dimensions 50 × 60–cm each; sampling 2400 Hz). The force plates were color-coordinated to match the walkway. For usable kinetic data, the landing had to be clean on one of these force plates. Controls were asked which leg they would consider as being their dominant leg or, if they did not know, to report the leg they would typically use to kick a ball, which was then taken as the dominant leg. Their nondominant leg was then used as a reference for the patient's minimal normal movement behavior, which is a conservative statistical approach. Before the SLH test, participants were required to perform a submaximal pretest consisting of a 40-cm forward hop with arms at the hips and a stable single-leg landing. Adequate hip, knee and ankle motion in the sagittal and frontal planes of this pretest was visually inspected by 2 testers according to the return to activity criteria for evaluating the movement quality of SLHs defined by Keller et al.^26^ Only if these return to activity criteria were met, patients were asked to perform actual SLH testing of maximum forward distance, where they were asked to stand on 1 leg, hop as far as possible without restriction of arm movement, land stably on the same leg, and maintain this position for at least 2 seconds (valid SLH). Participants had 4 trials per leg (in alternating leg order) to reach their maximum SLH distance with a stable landing. Sufficient rest between trials of the same leg was guaranteed in part by the time needed to prepare the technical system for the next trial. To increase the chance of landing on the force plates, the first trial was performed away from the force plates, and a tape was placed on the floor as a starting line. Participants then performed maximum trials from this tape toward the force plates, but were unaware of the force plates (same color as and embedded in the walkway). If <3 valid SLH attempts were made, or if participants felt that they had not yet reached the maximum distance, a maximum of 2 additional attempts per leg were allowed.

### Data Collection and Preparation

Motion analysis of the SLH was performed using a camera motion capture system (VICON; sampling 240 Hz) and synchronized force plates. Marker data of an upright standing trial were recorded to define joint coordinate systems (Conventional Gait Model marker set).^34^ Marker trajectories were processed (labeled, gap filled, and filtered [Woltring filter^65^]) using Nexus (VICON). Sagittal plane kinematics and kinetics of the hip, knee and ankle and frontal plane kinematics and kinetics of the hip and knee were calculated according to the model.^34^ Kinetic data (joint moments and power, and vertical GRF) were filtered (4^th^-order zero-lag lowpass Butterworth, cut off 15 Hz)^40^ and normalized to participant body mass (moments: Nm/kg, expressed as external moments) or to body weight (GRF: BW).

### Data Analysis

Data analysis was performed using MATLAB (R2020b, The MathWorks Inc). Data from the valid SLH with the greatest distance for each participant and leg during the landing phase, defined as the time interval from initial contact (IC) to maximum knee flexion, were analyzed.^40^ Discrete parameters in sagittal plane hip, knee and ankle kinematics and kinetics, and frontal plane hip and knee kinematics and kinetics, and vertical GRF data during the landing phase were calculated for each leg and group according to previous studies on patients after ACLR—values at the IC and peaks of joint angles^24,27,36^; peaks of joint moments^24,27,36^; joint work contribution to total work (Equation 1)^29^; peak vertical GRF^24,36^; and loading rate (Equation 2).^49^

$$\mathrm{work}\;\mathrm{contributio}n_{\mathrm{hip}/\mathrm{knee}/\mathrm{ankle}}=\;\frac{\int \mathrm{neg}.\;\mathrm{powe}r_{\mathrm{hip}/\mathrm{knee}/\mathrm{ankle}}}{\mathrm{sum}(\int \mathrm{neg}.\;\mathrm{powe}r_{\mathrm{hip}}+\int \mathrm{neg}.\;\mathrm{powe}r_{\mathrm{knee}}+\int \mathrm{neg}.\;\mathrm{powe}r_{\mathrm{ankle}})\;}*100$$

[2] $$\mathrm{loading}\;\mathrm{rate}=\;\frac{\mathrm{peak}\;\mathrm{verical}\;\mathrm{GRF}}{\mathrm{time}\;(\;\mathrm{peak}\;\mathrm{vertical}\;\mathrm{GRF}-\mathrm{IC})}$$

**Figure 1.**
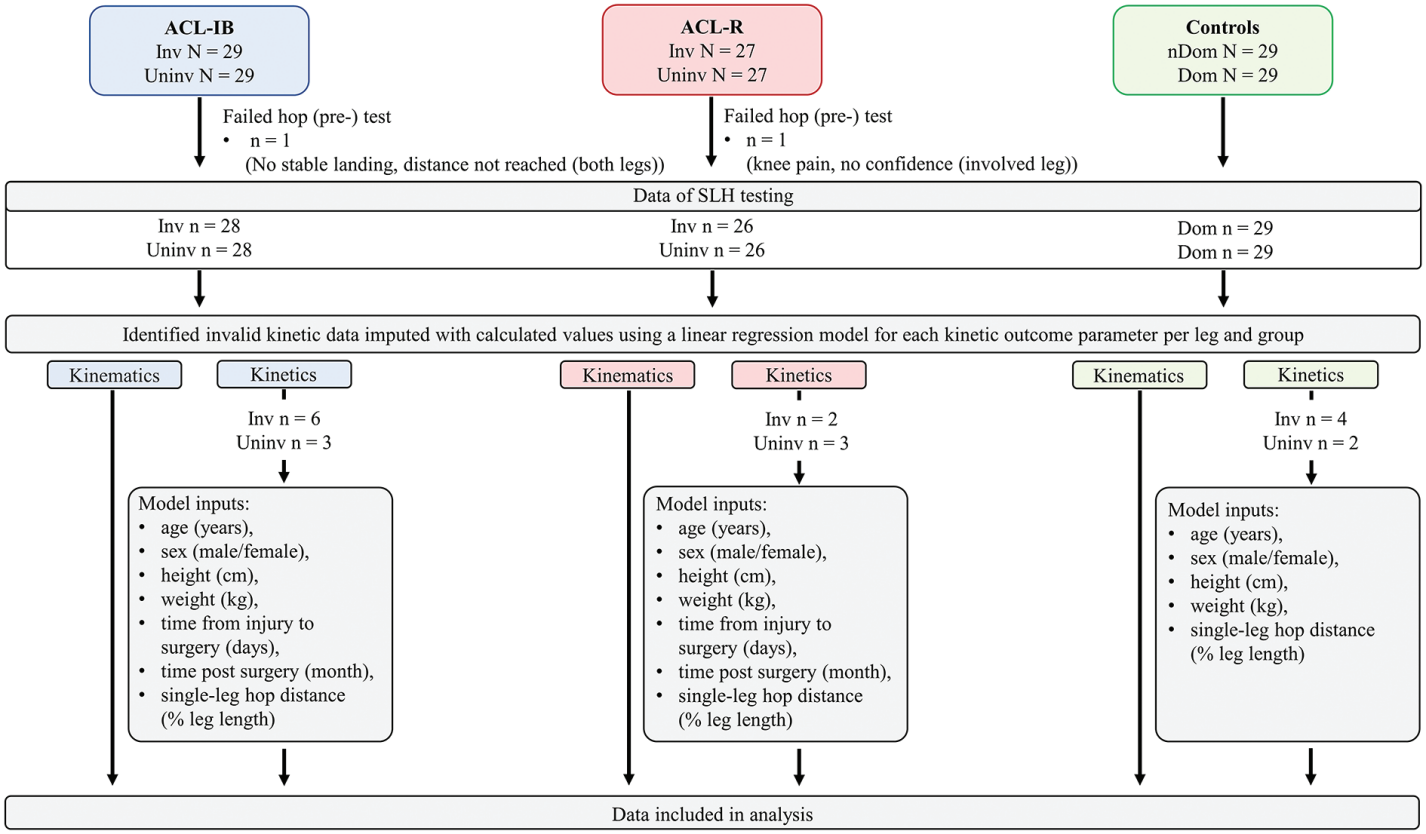
Overview of our collected SLH data set with fully usable kinematic data as well as usable and unusable kinetic data, including input parameters for the linear regression model for each leg and group that were used to impute data for cases with unusable kinetic data.

Only participants who were able to perform the SLH were considered for analysis. If the farthest valid SLH was not landed entirely on one of the force plates, the kinetic data were considered unusable, as correct kinetic analysis requires full landing of the foot on 1 force plate. For trials with usable kinetic data, the IC was defined as the time when the vertical GRF exceeded 10 N. For trials with unusable GRF data (stable SLH landing, but not landing on a single force plate), the IC was determined visually from the marker data in Nexus (VICON). In a subsample of trials with usable kinetic data (patients and controls, N = 25), an offset of 2 ± 2 ms (mean ± SE, corresponding to 1 to 2 frames) was observed between the visually determined and the force plate determined IC, which we considered to be negligible. To maintain statistical power for the kinetic data, for cases of valid SLHs with unusable kinetic data, we imputed the kinetic outcome parameters (ie, local peaks of joint moments; joint work contribution to total work, peak vertical GRF; and loading rate) using a linear regression model for each group and leg.^20,67^ The model was fitted to the fully available usable kinetic data set of valid SLHs (all landings on a single force plate) based on anthropometric (age, sex, height, weight), injury-related (time injury to surgery, time of follow-up testing), and hop-specific (maximum SLH distance) input parameters for each group and leg and then used to predict the discrete kinetic data for the cases with unusable kinetic data (Figure 1). We did not use the second farthest SLH that had been successfully landed on the force plate because the horizontal hop distance, which was shorter in the second farthest SLHs, can affect the landing kinematics or kinetics of the ankle, knee, and hip.^1,57^ To justify our approach and assess the robustness of our findings to the effect of imputation, we performed a sensitivity analysis comparing our results between the usable kinetic data set (excluding unusable kinetic cases) and the kinetic data set with usable and imputed kinetic data.

### Statistical Analysis

According to the statistical power calculation of the umbrella project (based on the main outcomes of the umbrella project; ie, functional parameters of balance and proprioception), at least 28 patients per group were needed to achieve a statistical power of 80%, with an alpha level of 5%.^43^ Statistical analyses were performed using MATLAB (R2020b; The MathWorks Inc).

For our primary aim, we compared the discrete parameters between the involved and uninvolved legs in patients after ACL-IB (Comparison I: within-patient difference in the ACL-IB group) and after ACLR (Comparison II: within-patient difference in the ACLR group) using a paired *t* test, respectively, and we also compared the discrete parameters between the involved leg in patients and the nondominant leg in controls (Comparison III: between-group differences including involved ACL-IB patients and controls, involved ACLR patients and controls, and involved ACL-IB and involved ACLR patients) using 1-way analysis of variance with post hoc Tukey test to assess statistical significance (*P* < .05).

For our secondary aim, we calculated the 95% CIs of the within-patient differences in the 2 ACL groups and the 95% CIs of the between-group differences. As described above, we identified landing biomechanic parameters falling outside the typical range observed in controls (see Introduction) by calculating the 95% CI of the within-control difference (the nondominant and dominant leg) as a reference interval and comparing the 95% CI of significant within-patient and between-group differences with the reference interval.^31,45^ Based on our assumption that controls do not have relevant differences in their legs, we considered leg differences that fall within this range to be, at most, irrelevantly worse or better.^31,52^ In contrast, significant leg differences with CIs that fall outside the reference interval are likely to be greater than typically seen in sex- and age-matched controls and were therefore considered not only significant, but also atypical with potential clinical relevance and/or meaning.^31,45^

## Results

Overall, we included 29 patients who underwent ACL-IB, 27 patients who underwent ACLR (N = 4 with additional gracilis tendon as 4-folded semitendinosus graft diameter alone was <7 mm), and 29 healthy controls (Table 1). To achieve an appropriate age matching between patients and controls (±4 years), we had to deviate from the published protocol and include 5 patients who had ACLR from 2 other medical centers. No professional athletes were represented in either patient group. The groups did not differ in their characteristics, concomitant injuries, follow-up time, or maximum forward hop distance (Table 1), as previously reported in other manuscripts related to the umbrella project.^7,44^ As early surgery is recommended for this procedure,^58^ patients were operated on earlier after ACL-IB than patients who had ACLR.^44^

**Table 1:** Characteristics and Hop Performance of Patients and Controls*^*a*^*

Parameter	ACL-IB Group (n = 29)		ACLR Group (n = 27)		Control Group (n = 29)	
Characteristics						
Sex, male/female	13/16		13/14		13/16	
Age, y	36.8 (10.6)		37 (10.7)		37 (10.7)	
Body height, cm	172.2 (7.8)		170.5 (7.4)		172.6 (10.8)	
BMI,*^*b*^* kg/m^2^	25.5 [21.2;26.5]*^*b*^*		24.5 [21.9;27.5]*^*b*^*		23.1 [20.4;24.9]*^*b*^*	
Injury						
Time from injury to surgery, days	20 [15-25]*^*b*^*		28 [14-56]*^*b*^*			
Operated leg, left/right	13/16		12/15			
Concomitant injuries and surgeries	23 (79)		24 (89)			
Follow-up after surgery,*^*b*^* months	24.4 [23.6;27.2]*^*b*^*		24.2 [23.6;24.9]*^*b*^*			
TAS at follow-up*^*b*^*	4 [4;6]*^*b*^*		4 [4;5]*^*b*^*		4[3-5]*^*b*^*	
Hop performance	Inv	UnInv	Inv	UnInv	NDom	Dom
Maximal SLH distance (%body height)	64 (22.7)	68.1 (20.8)	62.8 (15.3)	68.6 (15.8)	68.7 (15.4)	70 (14.8)

Data are retrieved from linked studies.^7,44^

a Values other than the number of participants (n) are given as mean (SD), unless otherwise indicated.

b Values are given as median [25;75] percentile, as data were not normally distributed according to the Shapiro-Wilk test (*P* < .05).

ACL-IB, patients after InternalBrace-augmented ACL repair; ACLR, patients after anterior cruciate ligament reconstruction; Dom, dominant leg; Inv, involved leg; NDom, nondominant leg; SLH, single-leg hop; TAS, Tegner Activity Score; UnInv, contralateral uninvolved leg.

One patient in the ACL-IB group (no stable landing for both legs) and 1 patient in the ACLR group (knee pain and no confidence in the involved leg) did not pass the SLH pretest, as previously reported^7^ (Figure 1). All other patients and controls who passed the pretest reported no knee pain during SLH. Kinetic data (moments, power, and GRF) were not usable and thus imputed in 6 involved and 4 uninvolved legs of patients in the ACL-IB group, in 2 involved and 3 uninvolved legs of patients in the ACLR group, and 4 nondominant and 2 dominant legs of the control group (imputation for approximately 13% of kinetic data) (Figure 1). The results of the sensitivity analysis are provided in the supplements (Supplemental Table S1) and show only minor changes in the mean, standard deviation, and confidence interval of the differences in the discrete kinetic parameters with imputed data for unusable kinetics. While the statistical within-patient comparisons in ACLR and the between-group comparison were not affected by imputation, in the ACL-IB group, imputation of kinetic data changed the significance of the within-patient difference between the legs for peak hip flexion moment, meaning that this specific statistical result should be interpreted with caution. Nonetheless, imputation of kinetic data for the cases with unusable kinetic data did not change the interpretation of our results, and, overall, the results of our sensitivity analysis support our approach of not excluding complete data sets merely because the GRF was not usable due to the landing position. Our post hoc power calculations revealed that all statistically significant results (*P* < .05) had a power of >90%.

The mean landing time was between 0.19 and 0.21 seconds for all groups and legs and did not differ significantly in any comparison (*P*≥ .602; Appendix Table A1).

### Within-Patient Differences

**Figure A1.**
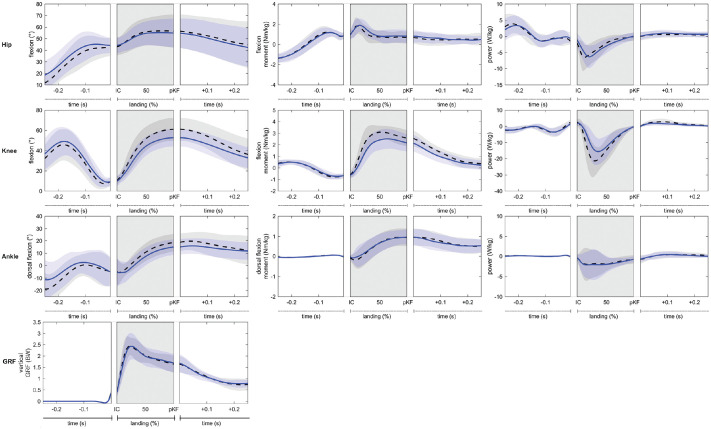
The mean (solid line) and 1 SD (shaded area) of trajectories of sagittal plane hip, knee, and ankle kinematics and kinetics, as well as the vertical GRF in BW of the involved (solid blue) and contralateral uninvolved (dashed black) legs in patients after ACL-IB. Trajectories were time-normalized only during landing (grey area, IC to PKF, 0%-100% landing). ACL-IB, InternalBrace-augmented anterior cruciate ligament repair; BW, body weight; GRF, ground reaction force; IC, initial contact; PKF, peak knee flexion.

In the ACL-IB group, the involved leg had significantly less peak knee flexion (mean difference [MD], −8.3°± 12°; effect size [Cohen’s *d*] = −0.85; *P* = .001), less peak ankle dorsiflexion (MD, −4.1°± 7.1°; *d* = −0.64; *P* = .005), a higher peak hip (MD, 0.30 ± 0.68 Nm/kg; *d* = 0.45; *P* = .025), and a lower peak knee flexion moment (MD, −0.60 ± 0.55 Nm/kg; *d* = −0.72; *P* < .001) compared with the uninvolved leg (Appendix Table A1 and Appendix Figure A1). In addition, the work contribution of the hip was significantly greater (MD, 7% ± 8.4%; *d* = 1.16; *P* < .001) and that of the knee was significantly smaller (MD, −9.5% ± 14.8%; *d* = −0.88; *P* = .002) in the involved leg compared with the uninvolved leg (Appendix Table A1).

**Figure A2.**
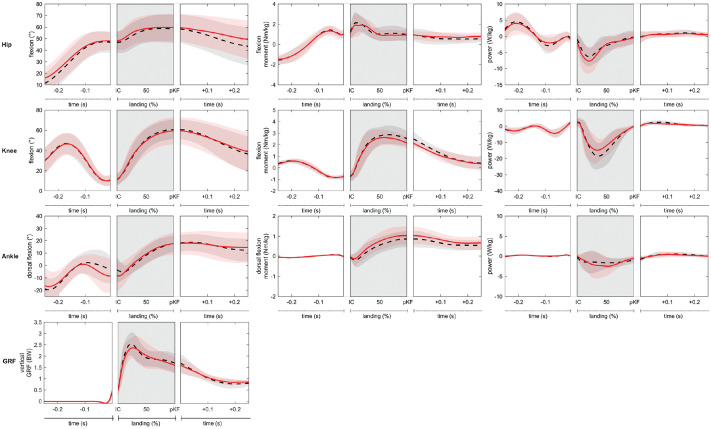
The mean (solid line) and 1 SD (shaded area) of trajectories of sagittal plane hip, knee, and ankle kinematics and kinetics, as well as the vertical GRF in BW of the involved (solid blue) and contralateral uninvolved (dashed black) legs in patients after ACLR. Trajectories were time-normalized only during landing (grey area, IC to pKF, 0%-100% landing). ACLR, anterior cruciate ligament reconstruction; BW, body weight; GRF, ground reaction force; IC, initial contact; PKF, peak knee flexion.

In the ACLR group, the involved leg had a significantly higher peak ankle dorsiflexion moment (MD, 0.17 ± 0.39 Nm/kg; *d* = 0.47; *P* = .039) compared with the uninvolved leg (Appendix Table A1 and Appendix Figure A2). In addition, the hip work contribution was significantly higher (MD, 4.8% ± 9%; *d* = 0.56; *P* = .011), whereas the knee work contribution was significantly lower (−7.1% ± 12.70%; *d* = −0.81; *P* = .009) in the involved leg compared with the uninvolved leg (Appendix Table A1).

No significant within-patient differences were found for frontal plane parameters in the 2 ACL groups (Appendix Table A1; Supplemental Figures S1 and S2).

### Between-Group Differences

**Figure A3.**
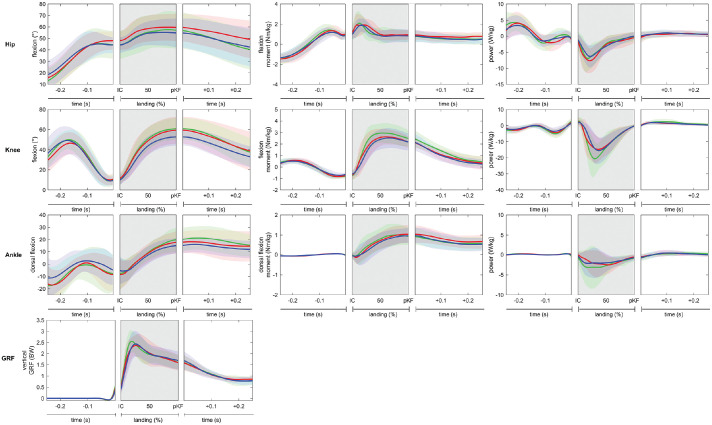
The mean (solid line) and 1 SD (shaded area) of trajectories of sagittal plane hip, knee, and ankle kinematics and kinetics, as well as the vertical GRF in BW of patients and controls. The involved leg in patients after ACL-IB (blue). The involved leg in patients after ACLR (red). The nondominant leg in healthy controls (green). Trajectories were time-normalized only during landing (grey area, IC to PKF, 0%-100% landing). ACL-IB, InternalBrace-augmented anterior cruciate ligament repair; ACLR, anterior cruciate ligament reconstruction; BW, body weight; GRF, ground reaction force; IC, initial contact; PKF, peak knee flexion.

Landing biomechanics did not differ significantly between the involved legs of the patients in the ACL-IB and ACLR groups (Appendix Table A1; *P*≥ .096). The ACL-IB group had less peak knee flexion (MD, −7.8°± 11°; *d* = −0.83; *P* = .019) and lower peak knee flexion moments (MD, −0.50 ± 0.85 Nm/kg; *d* = −0.71; *P* = .020) in the involved leg compared with the nondominant leg of the control group (Appendix Table A1 and Appendix Figure A3), while the ACLR group had a significantly higher hip contribution (MD, 4.9% ± 11.0%; *d* = 0.67; *P* = .019) in the involved leg compared with the nondominant leg of the control group (Appendix Table A1).

No significant between-group differences between the involved legs of patients and the nondominant leg of controls were found for frontal plane parameters (Appendix Table A1 and Supplemental Figure S3).

### Within-Patient and Between-Group Differences Greater Than Typical Symmetry Variations

Of the statistically significant within-patient differences in the 2 ACL groups, only the 95% CI of the peak knee flexion and peak knee flexion moment in the ACL-IB group were outside the reference interval (no overlap with the 95% CI of within-control difference; Appendix Table A1).

Of the statistically significant between-group differences, only the 95% CI of the difference in the peak knee flexion moment between the involved leg in the ACL-IB group and the nondominant leg in the control group was outside the reference interval (no overlap with the 95% CI of the within-control difference; Appendix Table A1).

The mean (±1 SD) trajectories of the kinematic and (not imputed) kinetic data during landing for the sagittal plane are shown in Appendix Figures A1 to A3. Frontal plane trajectories of leg comparisons are provided in Supplemental Figures S1 to S3.

## Discussion

Our primary aim was to investigate differences in sagittal and frontal plane lower limb kinematics and kinetics and vertical GRF during SLH landing between the legs in patients 2 years after ACL-IB and in patients 2 years after ACLR, as well as between the involved legs of patients and the nondominant leg of controls. In addition, our secondary aim was to compare the resulting 95% CIs of the leg differences in patients with the 95% CI of the leg differences in sex- and age-matched controls to identify leg changes that were greater than the typical symmetry variations seen in controls and therefore might be clinically relevant.

As expected, and stated in our hypothesis, sagittal and frontal kinematics and kinetics did not differ between the involved legs of patients 2 years after surgery. However, we observed differences in lower limb biomechanics between the involved and uninvolved legs in both patient groups, and between the involved legs of patients and the nondominant leg of controls in the sagittal plane only. Interestingly, after ACL-IB, both kinematics and kinetics differed significantly between the involved and uninvolved legs and compared with controls, whereas after ACLR, only kinetics differed significantly between the involved and uninvolved legs and compared with controls. In addition, patients showed altered landing biomechanics after only ACL-IB that were greater in magnitude than the typical symmetry variations seen in the legs in our controls (exceeded 95% CI of within-control difference).

To our knowledge, no data are available on knee biomechanics during dynamic, athletic tasks in patients after ACL-IB. Two meta-analyses investigating leg biomechanics of hop landings in patients between 27 weeks and 31 years after ACLR reported a lower peak knee flexion angle and moment in the involved compared with the uninvolved leg and compared with the leg of healthy controls.^24,27^ Interestingly, we observed similar differences in the ACL-IB group but not in the ACLR group (neither between their legs, nor compared with controls). Differences between our study and previously reported study results after ACLR may be related to the inclusion of multiple graft types for reconstruction, multiple hop landing tasks (reactive vs nonreactive; single vs double leg landing), different postoperative measurement time points, and/or different activity levels of the patients.^24,27^

Only in the ACL-IB group, the significant differences between the legs (involved vs uninvolved) in peak knee flexion angle and moment were with a 95% probability outside the typical range as defined by our controls. This was also true for the peak knee flexion moment in the ACL-IB group (involved leg) compared with controls. In both comparisons, the magnitudes, effect sizes, and confidence intervals of the differences in peak knee flexion were comparable, and the magnitude (approximately 8°) exceeded another reported threshold for a clinically important difference (>3°), also based on controls.^11^ However, the applicability of this reported threshold to our results may be limited because this value was obtained during walking.^11^ The likewise comparable differences and effect sizes in peak knee flexion moment in the involved leg in the ACL-IB group compared with the uninvolved leg and compared with the nondominant leg in controls reinforce relevant biomechanical deviations in the ACL-IB group and suggest that the observed changes in knee parameters in the ACL-IB group during SLH landing are caused by abnormal deviation of the involved leg and not the uninvolved leg 2 years after surgery. In this context, the lower ankle dorsiflexion in the involved leg after ACL-IB may be related to the lower peak knee flexion to still support a stable upright landing position.

External knee flexion moments are balanced by internal knee extension moments generated primarily by the quadriceps muscles that apply shear forces to the knee via the patellar tendon.^39^ This can stress the ACL and/or cause anterior tibia translation, which is primarily limited by the ACL.^8^ Although the quadriceps muscle has a greater lever arm to pull the tibia anteriorly at smaller knee flexion angles,^39^ less knee flexion during landing results in a smaller lever arm for the external knee flexion moment. With an unchanged GRF, a lower quadriceps muscle force is required to counteract the external knee flexion moment. Lower external knee flexion moments and/or quadriceps strength have been reported in the involved legs of patients after ACLR who had less knee flexion or less knee flexion excursion during landing of a standardized SLH^55^ or crossover hop.^32^ In addition, a strategy of less knee flexion to reduce anterior tibia translation during SLH has recently been observed in healthy individuals with passive anterior knee laxity.^25^ Therefore, it appears that after ACL-IB, patients may have a stiffer knee landing strategy to potentially stabilize the knee anteriorly and potentially reduce the demand on knee muscles during dynamic landings. However, other studies have reported a greater anterior tibia translation in patients after ACLR during forward SLH using different measurement techniques (biplane radiography,^10^ marker-based^47^), despite a lower knee flexion angle and/or external knee flexion moment in the involved leg compared with the healthy contralateral leg. Our analysis did not allow us to determine whether patients were able to achieve a normal or healthy anterior tibia translation with this strategy after ACL-IB. Regardless of the underlying mechanism, landings with less knee flexion have been reported to result in higher peak ACL forces compared with those with more knee flexion,^33^ and lower knee flexion angles have been associated with an increased risk of knee reinjury.^4,18^ Our results are consistent with the higher risk of rupture associated with patients after ACL-IB participating in high knee loading activities^22^ (eg, Tegner Activity Score >7). Therefore, the observed landing biomechanics in the ACL-IB group seem to be relevant and meaningful.

The changes in joint work contribution in patients may indicate a possible compensation by the hip for a lower contribution of the knee in the involved compared with the contralateral leg after both ACL surgeries. Only patients who underwent ACLR but not ACL-IB differed significantly from controls in this parameter. A meta-analysis including patients after ACLR showed that the involved knee absorbed less power than the uninvolved knee during hop landings.^27^ In addition, a higher hip and/or lower knee contribution in the involved leg in patients after ACLR compared with the uninvolved leg or with controls during hop tasks (ie, drop, vertical, or double limb jump) has been reported up to 2 years postoperatively.^5,30,46^ Considering that we observed a potential involved knee unloading in both groups, with a trend toward different kinematics after both surgeries in the involved leg (and compared with controls), there may be a different strategy for knee unloading between patients. However, this remains speculative, and such changes were also observed in some of our controls (overlapping CIs).

Our recent study of side-to-side differences in the same population of patients who had ACL-IB showed fewer leg differences during less dynamic walking^6^ compared with the highly dynamic SLH landing in patients after ACL-IB. It is possible that the dynamics of a task cause leg differences in the ACL-IB group to rise. The biomechanical results of this study and our previous findings during walking do not support the assumption of restored knee biomechanics compared with controls, as suggested in the literature.^51,59^ To our knowledge, there is no information on the in vivo characteristics of a healed and augmented ACL after repair in humans, and it is unknown whether this ACL fully regains the characteristics of a native ACL and is or will be naturally loaded. In a cadaveric study, human knees after ACL-IB did not achieve passive knee stability and ACL in situ forces of the native ACL knee, especially under anterior tibial loads.^21^ The characteristics and function of the ACL in our patients after ACL-IB may still be different from those of patients with an intact ACL—including a different response to loading or stretching—2 years postoperatively. Whether biomechanical ACL characteristics and function may still change beyond this time point is currently unknown. Some authors even suggest that the tape augmentation acts as a load-bearing device and therefore may impede “proper” healing.^2^ Consequently, it is suspected that the ACL may be subjected to altered stress, resulting in a different or even diminished tissue response.

Overall, both patient groups had significant differences in sagittal plane knee biomechanics during SLH landing between legs and between the involved leg and the nondominant leg of age- and sex-matched healthy controls 2 years after surgery. While our results are promising for dynamic but not overly demanding tasks in a moderately knee-loading active population after ACL-IB, evidence is lacking regarding the trade-off between the surgical benefit and potentially limited biomechanical knee function and stability in more complex and demanding activities and elite athletes. Possible associations between clinical, functional, and biomechanical parameters and implications of our results regarding the risk of reinjury or the onset of secondary knee joint degeneration after ACL-IB warrant further investigation.

### Limitations

This study has several limitations. Although the same rehabilitation program was prescribed in all patients regardless of their hospital origin, we were unable to monitor adherence to the rehabilitation program, which represents a limitation of our study. Our method of imputing unusable kinetic data using a linear regression model may have underestimated the variability in our kinetic data,^14,46^ resulting in a smaller kinetic confidence interval. Nonetheless, this approach was more favorable than the alternative of excluding all valid SLH data with unusable kinetic data, which would have also required excluding the usable kinematic data of this trial. This would have led to a smaller sample size, despite having this data. The use of larger force plates may help to overcome the problem of unusable kinetic data from valid SHL trials, which were not available in our laboratory at the time of data collection. The controls’ nondominant leg was used as a reference for normal movement behavior, which is a conservative statistical approach. While it might be expected that the differences would be greater compared with the dominant leg of controls, not all controls had a lower SLH performance in their nondominant leg, negating this assumption. The difference in time from injury to surgery between the ACL-IB and ACLR groups may have introduced a selection bias. However, addressing this issue is challenging, as early surgery is recommended for ACL repair, whereas early ACLR is typically not required or enforced. We were able to show that the 10% difference in concomitant injuries between our patient groups was not significant,^44^ but we did not analyze the potential effect of this difference on our outcome parameters. In addition, we did not consider the function of the surrounding knee muscles, which may play an important role in knee stability and control after surgery. The transferability of our results to other tasks is limited and may lead to different results between ACL-IB and ACLR groups.

## Conclusion

Changes in single-leg landing biomechanics in the uninvolved leg persisted 2 years after ACLR or ACL repair. This suggests that the initial ACL rupture causes changes that cannot be restored with surgery alone. Although comparable SLH landing biomechanics between the involved legs after both ACL surgeries were observed, only the biomechanical changes in the involved knee in patients after ACL-IB were deemed atypical compared with the uninvolved leg or with controls. Information and comparison with other tendon grafts, and analysis of movement execution during different (high-impact) tasks are warranted to explore the full benefits and possible limitations of ACL-IB.

## Supplemental Material

sj-pdf-1-ojs-10.1177_23259671251358386 – Supplemental material for Landing Biomechanics in Patients 2 Years After Augmented ACL Repair and 2 Years After Hamstring Autograft ACL Reconstruction Compared With Controls Supplemental material, sj-pdf-1-ojs-10.1177_23259671251358386 for Landing Biomechanics in Patients 2 Years After Augmented ACL Repair and 2 Years After Hamstring Autograft ACL Reconstruction Compared With Controls by Linda Bühl, Sebastian Müller, Corina Nüesch, Florian Samuel Halbeisen, Annegret Mündermann and Christian Egloff in Orthopaedic Journal of Sports Medicine

sj-pdf-2-ojs-10.1177_23259671251358386 – Supplemental material for Landing Biomechanics in Patients 2 Years After Augmented ACL Repair and 2 Years After Hamstring Autograft ACL Reconstruction Compared With Controls Supplemental material, sj-pdf-2-ojs-10.1177_23259671251358386 for Landing Biomechanics in Patients 2 Years After Augmented ACL Repair and 2 Years After Hamstring Autograft ACL Reconstruction Compared With Controls by Linda Bühl, Sebastian Müller, Corina Nüesch, Florian Samuel Halbeisen, Annegret Mündermann and Christian Egloff in Orthopaedic Journal of Sports Medicine

## Appendix

**Table A1:** Mean (±1 SD) of Sagittal and Frontal Leg Biomechanics in Patients and Controls During Landing*^*a*^*

Parameter	ACL-IB Group		ACLR Group		Control Group		Within-Patient Comparison: Involved vs Uninvolved		Between-Group Comparison: Involved Patients vs Nondominant Controls			Reference Interval for Atypical Differences
	Involved	Uninvolved	Involved	Uninvolved	Nondominant	Dominant	ACL-IB	ACLR	ACL-IB vs Controls	ACLR vs Controls	ACL-IB vs ACLR	Controls
	Mean (SD)	Mean (SD)	mean (SD)	Mean (SD)	Mean (SD)	Mean (SD)	95% CI	95% CI	95% CI	95% CI	95% CI	95% CI
Landing phase	0.21 (0.20)	0.19 (0.06)	0.20 (0.12)	0.20 (0.12)	0.21 (0.14)	0.21 (0.10)	0.21 (0.10)	[−0.05 to 0.09]	[−0.10 to 0.10]	[−0.11 to 0.09]	[−0.09 to 0.11]	[−0.03 to 0.03]
Sagittal plane angles, deg												
Hip flexion												
IC	44.4 (6.8)	43.4 (7.7)	48.3 (8.2)	46.9 (9.4)	44.5 (12.2)	44 (9.3)	[−1.1 to 3.1]	[−1.1 to 3.4]	[−6.1 to 5.9]	[−2.3 to 9.8]	[−10 to 2.3]	[−2 to 3]
Peak	56.3 (11.3)	58.1 (14.1)	61.2 (11.9)	59.9 (12.1)	59.1 (16.2)	57.2 (14.6)	[−5.6 to 1.9]	[−1.3 to 4.2]	[−11.2 to 5.7]	[−6.5 to 10.8]	[−13.6 to 3.8]	[−1 to 4.7]
Knee flexion												
IC	10.6 (4.9)	11.7 (4.0)	12.3 (5.9)	12 (5.7)	11.6 (5.3)	11.5 (5)	[−3 to 0.8]	[−2.2 to 1.3]	[−4.4 to 2.4]	[−2.8 to 4.1]	[−5.2 to 1.8]	[−1.5 to 1.7]
Peak	52.7 (8.6)	61.1 (11.3)	59.4 (13.4)	60.6 (9.6)	60.8 (10.7)	59.6 (9.6)	**[−13 to −3.7]** *^*b*^*	[−5 to 3.2]	**[−15.1 to –1.1]**	[−8.6 to 5.6]	[−13.8 to 0.5]	[−1.7 to 4.2]
Ankle dorsiflexion												
IC	−5.5 (10.5)	−5.7 (10)	−8.4 (11.8)	−4.1 (9.5)	−7.5 (12.4)	−6.1 (12.5)	[−3.7 to 4.1]	[−9 to 0.3]	[−5.3 to 9.4]	[−8.3 to 6.6]	[−4.7 to 10.4]	[−6.3 to 3.4]
Peak	15.1 (6.2)	19.2 (6.9)	17.7 (9.1)	18 (6.7)	20 (9.6)	19.3 (7.7)	**[−6.9 to −1.3]**	[−3.6 to 3.9]	[−10.2 to 0.5]	[−7.7 to 3.2]	[−8.1 to 2.9]	[−2.4 to 3.8]
Sagittal plane moments, N m kg^-1^												
Hip flexion												
Peak	2.45 (0.61)	2.15 (0.73)	2.67 (0.79)	2.69 (0.73)	2.66 (0.74)	2.52 (0.77)	**[0.04 to 0.56]**	[−0.27 to 0.23]	[−0.67 to 0.24]	[−0.45 to 0.47]	[−0.69 to 0.24]	[−0.12 to 0.40]
Knee flexion												
Peak	2.81 (0.79)	3.33 (0.64)	3.02 (0.68)	3.21 (0.60)	3.35 (0.72)	3.20 (0.72)	**[−0.72 to −0.31]** ^*b*^	[−0.49 to 0.13]	**[−1 to −0.07]** ^*b*^	[−0.80 to 0.15]	[−0.69 to 0.27]	[−0.06 to 0.35]
Ankle dorsiflexion												
Peak	1.04 (0.36)	1.05 (0.41)	1.11 (0.39)	0.94 (0.34)	1.10 (0.51)	1.17 (0.48)	[−0.20 to 0.19]	**[0.01 to 0.32]**	[−0.33 to 0.21]	[−0.27 to 0.28]	[−0.34 to 0.21]	[−0.20 to 0.08]
Frontal plane angles, deg												
Hip abduction												
IC	−10.3 (6.7)	−9 (5.6)	−7.8 (5.1)	−8.5 (6.7)	−10.6 (8.1)	−10.3 (6.7)	[−4.1 to 1.5]	[−1.5 to 2.9]	[−4 to 4.6]	[−1.6 to 7.1]	[−6.9 to 1.9]	[−2 to 1.6]
Peak	4.4 (6.3)	1.8 (6.1)	4.4 (6.7)	1.5 (7)	1.6 (7.2)	0.8 (5.6)	[−0.3 to 5.5]	[−0.6 to 7.3]	[−1.5 to 7.1]	[−1.6 to 7.2]	[−4.4 to 4.4]	[−2 to 3.6]
Knee abduction												
IC	0.1 (3.1)	0.6 (2.5)	1.2 (3.5)	2.1 (2.9)	0.2 (3.7)	−0.6 (4.5)	[−1.5 to 0.6]	[−2.2 to 0.5]	[−2.2 to 2.1]	[−1.2 to 3.2]	[−3.3 to 1.2]	[−0.6 to 2.2]
Peak	3.5 (4)	5.3 (4.4)	5.3 (4.6)	6.4 (4.4)	5.8 (4.6)	5.8 (6.1)	[−3.5 to 0]	[−2.5 to 1]	[−5.1 to 0.4]	[−3.4 to 2.3]	[−4.7 to 1]	[−2.2 to 2.2]
Frontal plane moments, N m kg^-1^												
Hip adduction												
Peak	1.48 (0.39)	1.51 (0.51)	1.55 (0.30)	1.55 (0.31)	1.51 (0.43)	1.67 (0.52)	[−0.21 to 0.16]	[−0.16 to 0.17]	[−0.27 to 0.21]	[−0.20 to 0.29]	[−0.32 to 0.17]	[−0.36 to 0.03]
Knee adduction												
Peak	0.69 (0.30)	0.76 (0.27)	0.83 (0.25)	0.89 (0.28)	0.82 (0.25)	0.98 (0.40)	[−0.20 to 0.07]	[−0.18 to 0.06]	[−0.30 to 0.04]	[−0.17 to 0.18]	[−0.31 to 0.03]	[−0.29 to −0.03]
Work contribution, % of overall work												
Hip	25.5 (5.4)	18.5 (6.6)	27.3 (9)	22.5 (8.2)	21.9 (7)	20.5 (7.4)	**[3.7 to 10.3]**	**[1.2 to 8.5]**	[−1 to 8.2]	**[0.7 to 10.1]**	[−6.5 to 2.9]	[−2.8 to 5.7]
Knee	61.2 (11.2)	70.8 (10.5)	59.5 (9.8)	66.6 (7.7)	64 (8.9)	65.8 (9)	**[−15.3 to −3.8]**	**[−12.2 to −2]**	[−9.1 to 3.6]	[−10.9 to 2]	[−4.8 to 8.2]	[−6 to 2.3]
Ankle	13.3 (11.9)	10.8 (8.9)	13.2 (9.2)	10.9 (7.2)	14.1 (9.1)	13.7 (8.6)	[−3.2 to 8.2]	[−2.1 to 6.6]	[−7.3 to 5.6]	[−7.5 to 5.6]	[−6.5 to 6.7]	[−2.8 to 3.7]
Vertical GRF												
Peak, BW	2.82 (0.44)	2.71 (0.36)	2.74 (0.35)	2.85 (0.35)	2.83 (0.37)	2.86 (0.36)	[−0.02 to 0.26]	[−0.24 to 0.03]	[−0.25 to 0.24]	[−0.33 to 0.17]	[−0.17 to 0.33]	[−0.16 to 0.09]
Loading rate, BW s^-1^	70.3 (24.7)	66.5 (21)	72.9 (21.6)	75.4 (21.5)	77.3 (20.6)	81.2 (24.1)	[−4.7 to 12.3]	[−10.9 to 6]	[−21.2 to 7.1]	[−18.9 to 10.0]	[−17.2 to 11.9]	[−10.1 to 2.3]

a Bold values indicate significant differences revealed by a paired *t* test in comparisons between the involved and uninvolved legs in patients, or comparisons of legs between groups using 1-way analysis of variance with a post hoc Tukey test (*P* < .05). ACL-IB, patients after InternalBrace-augmented ACL repair; ACLR, patients after anterior cruciate ligament reconstruction; BW, body weight; GRF, ground-reaction force; IC, initial contact.

b Significant differences are considered atypical, as their 95% CI does not overlap with the reference 95% CI of leg (side-to-side) differences in controls.
