# Supplementary material for: Landing Biomechanics in Patients 2 Years After Augmented ACL Repair and 2 Years After Hamstring Autograft ACL Reconstruction Compared With Controls
Source: Orthop J Sports Med. 2025 Jul 25;13(7):23259671251358386. doi: 10.1177/23259671251358386 (PMC12304590; doi:10.1177/23259671251358386)
Supplement: sj-pdf-1-ojs-10.1177_23259671251358386 – Supplemental material for Landing Biomechanics in Patients 2 Years After Augmented ACL Repair and 2 Years After Hamstring Autograft ACL Reconstruction Compared With Controls [file sj-pdf-1-ojs-10.1177_23259671251358386.pdf]

Supplemental Material

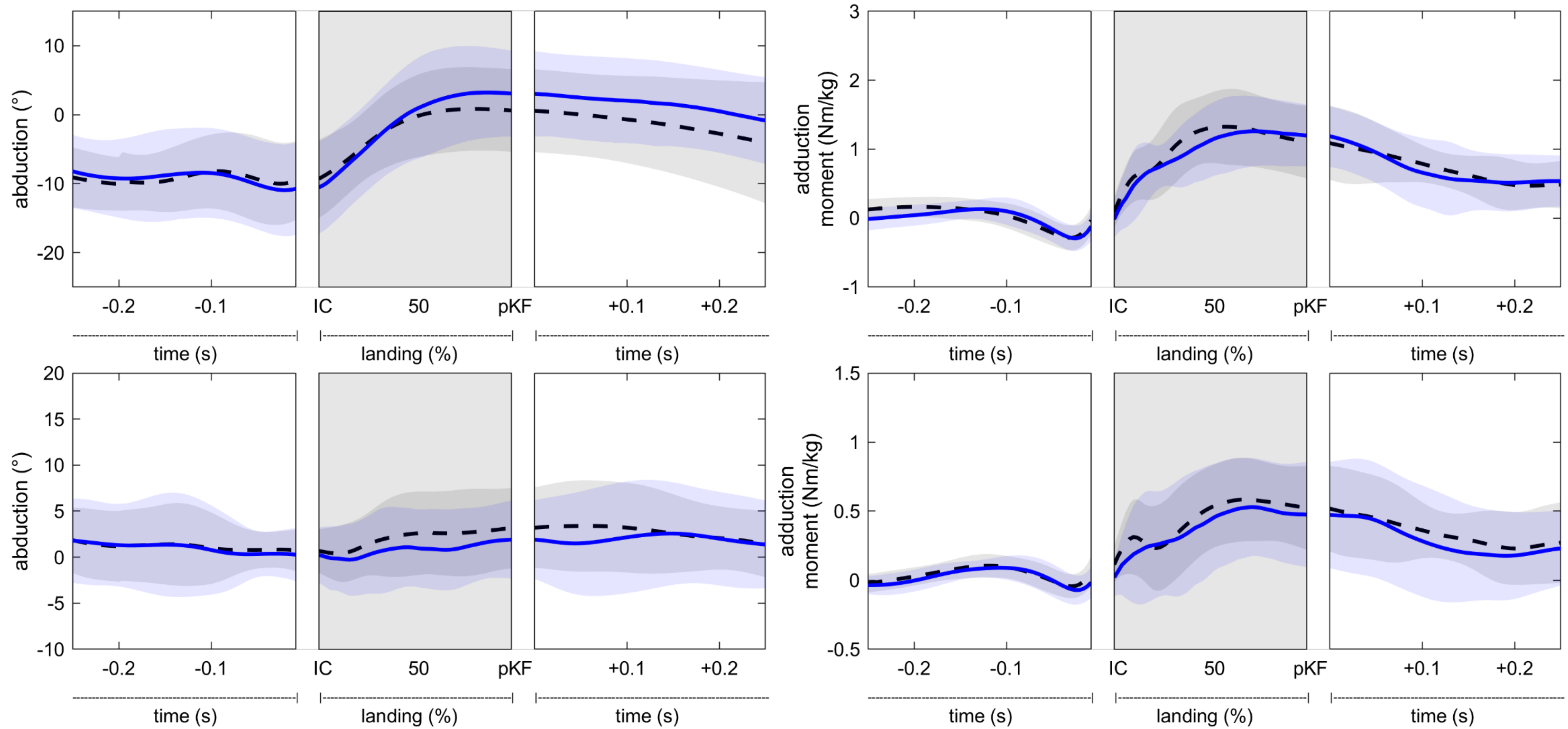

Figure S1: Mean (solid line) and 1 standard deviation (shaded area) of trajectories of frontal plane hip and knee kinematics and kinetics of the involved (solid blue) and the contralateral uninvolved (dashed black) leg in patients after InternalBrace^{TM}-augmented anterior cruciate ligament repair (ACL-IB). Trajectories were time normalized only during landing (grey area, initial contact (IC) to peak knee flexion (pKF), 0-100% landing).

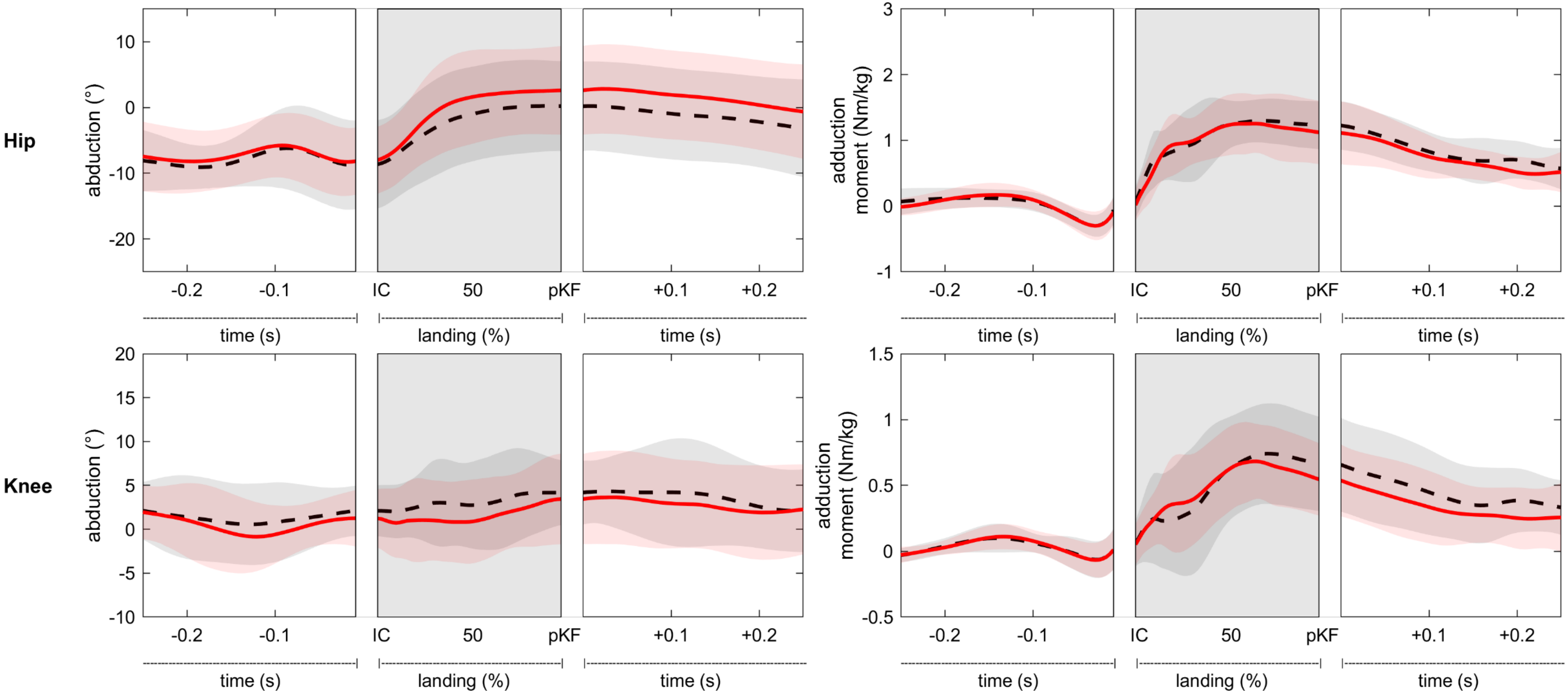

Figure S2: Mean (solid line) and 1 standard deviation (shaded area) of trajectories of frontal plane hip and knee kinematics and kinetics of the involved (solid red) and the contralateral uninvolved (dashed black) leg in patients after ACL reconstruction (ACL-R). Trajectories were time normalized only during landing (grey area, initial contact (IC) to peak knee flexion (pKF), 0-100% landing).

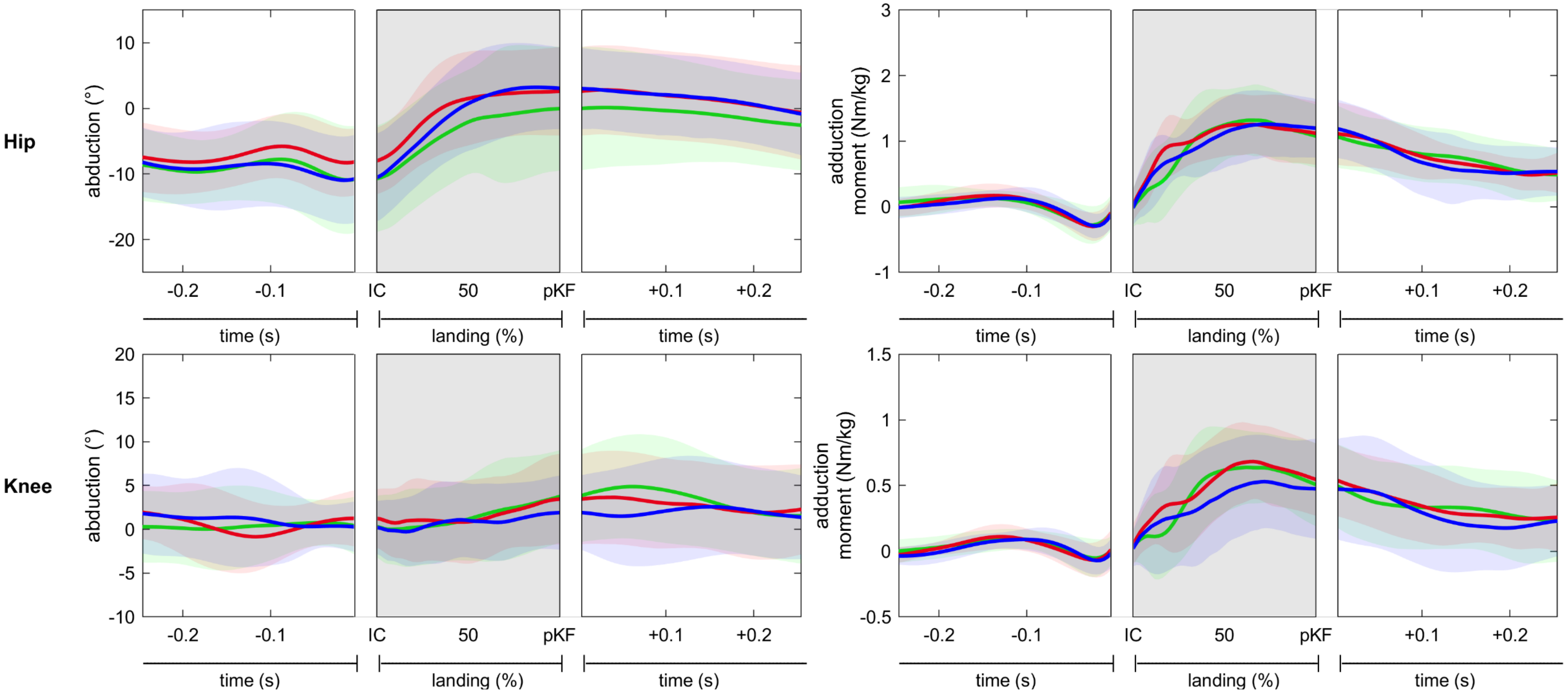

Figure S3: Mean (solid line) and 1 standard deviation (shaded area) of trajectories of frontal plane hip and knee kinematics and kinetics of the involved leg in patients after InternalBrace™-augmented anterior cruciate ligament repair (ACL-IB, blue), the involved leg in patients after ACL reconstruction (ACL-R, red) and the non-dominant leg in healthy controls (green). Trajectories were time normalized only during landing (grey area, initial contact (IC) to peak knee flexion (pKF), 0-100% landing).
