# Supplementary material for: Landing Biomechanics in Patients 2 Years After Augmented ACL Repair and 2 Years After Hamstring Autograft ACL Reconstruction Compared With Controls
Source: Orthop J Sports Med. 2025 Jul 25;13(7):23259671251358386. doi: 10.1177/23259671251358386 (PMC12304590; doi:10.1177/23259671251358386)
Supplement: sj-pdf-2-ojs-10.1177_23259671251358386 – Supplemental material for Landing Biomechanics in Patients 2 Years After Augmented ACL Repair and 2 Years After Hamstring Autograft ACL Reconstruction Compared With Controls [file sj-pdf-2-ojs-10.1177_23259671251358386.pdf]

Table S1: Results of the sensitivity analysis for kinetic parameters. Top: results of the analysis on the data set containing only trials with usable kinetic data (i.e. all isolated landings on a force plate); bottom: results of the analysis on the data set including trials with usable kinetic data and imputed kinetic data for cases where no isolated landing on a force plate was performed (approx. 13%). Prediction of kinetic data for imputation was performed by using a linear regression model fitted to the usable kinetic data set. Different statistical results for the analysis of the data set containing only usuable kinetics and the data set with usuable and iumputed kinetics are highlighted in grey.

	ACL-IB		*ACL-R*		*Controls*		*Within-subject comparison of the involved vs uninvolved leg in patients, and the non-dominan vs dominant leg in controls*									*Between-group leg difference between patients (involved leg ACL-IB vs uninvolved leg ACL-R vs non-dominant leg Controls)*									
	Involved	*Uninvolved*	*Involved*	*Uninvolved*	*Non-dominant*	*Dominant*	*leg difference in ACL-IB*			*leg difference in ACL-R*			*leg difference in Controls*				*ACL-IB vs Controls*			*ACL-R vs Controls*			*ACL-IB vs ACL-R*		
Parameter	*mean (SD)*	*mean (SD)*	*mean (SD)*	*mean (SD)*	*mean (SD)*	*mean (SD)*	*mean (SD)*	*95% CI*	*P-Value*	*mean (SD)*	*95% CI*	*P-Value*	*mean (SD)*	*95% CI*	*P-Value*	*ANOVA # P-Value*	*mean (SD)*	*95% CI*	*Tukey P-Value*	*mean (SD)*	*95% CI*	*Tukey P-Value*	*mean (SD)*	*95% CI*	*Tukey P-Value*
ANALYSIS OF DATA SET WITH USABLE KINETICS*																									
Peak vertical GRF (BW)	2.75 (0.40)	2.70 (0.37)	2.74 (0.36)	2.85 (0.37)	2.83 (0.38)	2.84 (0.36)	0.13 (0.36)	[-0.04;0.31]	0.119	-0.13 (0.33)	[-0.28;0.01]	0.075	-0.04 (0.26)	[-0.15;0.07]	0.501	0.673	-0.08 (0.53)	[-0.34;0.19]		-0.10 (0.45)	[-0.35;0.17]		0.03 (0.65)	[-0.26;0.28]	
Peak hip flexion moment (Nm/kg)	2.35 (0.55)	2.19 (0.75)	2.68 (0.81)	2.70 (0.80)	2.68 (0.80)	2.49 (0.79)	0.22 (0.61)	[-0.07;0.52]	0.129	-0.12 (0.64)	[-0.41;0.17]	0.388	0.15 (0.62)	[-0.12;0.41]	0.260	0.210	-0.30 (1.14)	[-0.85;0.18]		-0.05 (1.26)	[-0.50;0.50]		-0.25 (1.10)	[-0.85;0.18]	
Peak hip adduction moment (Nm/kg)	1.47 (0.41)	1.51 (0.53)	1.55 (0.31)	1.55 (0.33)	1.51 (0.46)	1.64 (0.52)	-0.09 (0.56)	[-0.36;0.18]	0.500	0.02 (0.43)	[-0.17;0.22]	0.823	-0.11 (0.52)	[-0.33;0.11]	0.302	0.780	0.04 (0.41)	[-0.32;0.24]		-0.03 (0.59)	[-0.23;0.31]		-0.15 (0.51)	[-0.36;0.20]	
Peak knee flexion moment (Nm/kg)	2.74 (0.74)	3.34 (0.66)	3.04 (0.67)	3.24 (0.64)	3.38 (0.71)	3.22 (0.69)	-0.57 (0.48)	**[-0.80;-0.34]**	**0.000**	-0.23 (0.61)	[-0.50;0.05]	0.104	0.15 (0.54)	[-0.08;0.37]	0.202	**0.011**	-0.49 (0.89)	**[-1.14;-0.14]**	**0.008**	-0.28 (1.09)	[-0.83;0.15]	0.220	-0.14 (1.01)	[-0.80;0.20]	0.327
Peak kneeadduction moment (Nm/kg)	0.67 (0.32)	0.76 (0.27)	0.83 (0.26)	0.89 (0.30)	0.83 (0.26)	0.97 (0.41)	-0.09 (0.39)	[-0.28;0.09]	0.310	-0.05 (0.32)	[-0.20;0.10]	0.475	-0.11 (0.34)	[-0.25;0.03]	0.124	0.102	-0.08 (0.36)	[-0.35;0.04]		0.00 (0.41)	[-0.19;0.19]		-0.16 (0.39)	[-0.35;0.04]	
Peak ankle dorsiflexion moment (Nm/kg)	1.04 (0.38)	1.05 (0.42)	1.11 (0.41)	0.93 (0.36)	1.13 (0.52)	1.17 (0.49)	-0.04 (0.56)	[-0.31;0.23]	0.737	0.20 (0.38)	**[0.03;0.37]**	**0.024**	-0.06 (0.39)	[-0.23;0.10]	0.426	0.783	0.04 (0.61)	[-0.40;0.22]		-0.08 (0.59)	[-0.32;0.29]		-0.04 (0.54)	[-0.38;0.25]	
Hip contribution (% overall work)	25.1 (5.8)	18.9 (6.6)	27.6 (8.8)	22.6 (8.9)	21.8 (7.4)	20.5 (7.6)	6.9 (8.9)	**[2.6;11.2]**	**0.003**	4.5 (9.4)	**[0.2;8.8]**	**0.042**	1.6 (11.2)	[-3.1;6.3]	0.486	**0.029**	2.9 (10.4)	[-1.9;8.6]	0.276	5.5 (10.5)	**[0.7;10.9]**	**0.022**	-2.6 (9.8)	[-7.7;2.8]	0.514
Knee contribution (% overall work)	60.0 (10.5)	70.2 (10.9)	59.7 (10.2)	66.7 (8.3)	64.0 (9.5)	65.6 (9.2)	-9.8 (12.1)	**[-15.6;-3.9]**	**0.002**	-7.4 (13.4)	**[-13.5;-1.3]**	**0.019**	-1.6 (10.9)	[-6.2;3.0]	0.481	0.254	-5.6 (11.2)	[-11.0;3.1]		-2.7 (14.4)	[-11.3;2.5]		-0.1 (16.8)	[-6.7;7.5]	
Ankle contribution (% overall work)	14.8 (10.7)	10.9 (9.0)	12.8 (9.1)	10.7 (7.8)	14.2 (9.8)	14.0 (8.8)	2.8 (13.3)	[-3.6;9.2]	0.363	2.9 (10.4)	[-1.8;7.7]	0.211	-0.0 (9.2)	[-3.9;3.9]	0.989	0.768	2.8 (13.0)	[-6.3;7.5]		-2.7 (12.8)	[-8.2;5.3]		2.7 (14.7)	[-4.9;9.0]	
Loading rate (BW/s)	66.6 (21.7)	65.7 (21.3)	73.5 (21.2)	76.1 (22.5)	77.6 (22.0)	79.9 (24.4)	2.4 (24.1)	[-9.2;14.0]	0.669	-3.4 (20.7)	[-12.6;5.7]	0.444	-1.8 (16.1)	[-8.7;5.0]	0.580	0.222	-11.5 (27.7)	[-26.2;4.1]		-4.3 (31.5)	[-18.9;10.8]		-3.3 (26.2)	[-22.3;8.3]	
ANALYSIS OF DATA SET WITH USABLE AND IMPUTED KINETIC DATA*																									
Peak vertical GRF (BW)	2.82 (0.44)	2.71 (0.36)	2.74 (0.35)	2.85 (0.35)	2.83 (0.37)	2.86 (0.36)	0.12 (0.35)	[-0.02;0.26]	0.084	-0.10 (0.32)	[-0.02;0.26]	0.112	-0.03 (0.32)	[-0.16;0.09]	0.577	0.680	0.01 (0.50)	[-0.25;0.24]		-0.06 (0.51)	[-0.33;0.17]		0.10 (0.66)	[-0.17;0.33]	
Peak hip flexion moment (Nm/kg)	2.45 (0.61)	2.15 (0.73)	2.67 (0.79)	2.69 (0.73)	2.66 (0.74)	2.52 (0.77)	0.30 (0.68)	**[0.04;0.56]**	**0.025**	-0.02 (0.62)	[-0.27;0.23]	0.884	0.14 (0.69)	[-0.12;0.40]	0.270	0.424	-0.20 (0.96)	[-0.67;0.24]		0.05 (1.21)	[-0.45;0.47]		-0.22 (1.13)	[-0.69;0.24]	
Peak hip adduction moment (Nm/kg)	1.48 (0.39)	1.51 (0.51)	1.55 (0.30)	1.55 (0.31)	1.51 (0.43)	1.67 (0.52)	-0.03 (0.48)	[-0.21;0.16]	0.768	0.01 (0.41)	[-0.16;0.17]	0.943	-0.16 (0.50)	[-0.36;0.03]	0.090	0.778	-0.02 (0.40)	[-0.27;0.21]		0.04 (0.60)	[-0.20;0.29]		-0.11 (0.52)	[-0.32;0.17]	
Peak knee flexion moment (Nm/kg)	2.81 (0.79)	3.33 (0.64)	3.02 (0.68)	3.21 (0.60)	3.35 (0.72)	3.20 (0.72)	-0.52 (0.53)	**[-0.72;-0.31]**	**0.000**	-0.18 (0.77)	[-0.49;0.13]	0.234	0.14 (0.54)	[-0.06;0.35]	0.165	**0.026**	-0.50 (0.85)	**[-1.00;-0.07]**	**0.020**	-0.29 (1.05)	[-0.80;0.15]	0.236	-0.23 (1.15)	[-0.69;0.27]	0.547
Peak kneeadduction moment (Nm/kg)	0.69 (0.30)	0.76 (0.27)	0.83 (0.25)	0.89 (0.28)	0.82 (0.25)	0.98 (0.40)	-0.07 (0.35)	[-0.20;0.07]	0.307	-0.06 (0.30)	[-0.18;0.06]	0.335	-0.16 (0.33)	**[-0.29;-0.03]**	**0.015**	0.096	-0.13 (0.30)	[-0.30;0.04]		0.01 (0.41)	[-0.17;0.18]		-0.17 (0.37)	[-0.31;0.03]	
Peak ankle dorsiflexion moment (Nm/kg)	1.04 (0.36)	1.05 (0.41)	1.11 (0.39)	0.94 (0.34)	1.10 (0.51)	1.17 (0.48)	-0.01 (0.51)	[-0.20;0.19]	0.949	0.17 (0.39)	**[0.01;0.32]**	**0.039**	-0.06 (0.37)	[-0.20;0.08]	0.353	0.826	-0.02 (0.59)	[-0.33;0.21]		-0.02 (0.58)	[-0.27;0.28]		-0.07 (0.50)	[-0.34;0.21]	
Hip contribution (% overall work)	25.5 (5.4)	18.5 (6.6)	27.3 (9.0)	22.5 (8.2)	21.9 (7.0)	20.5 (7.4)	7.0 (8.4)	**[3.7;10.3]**	**0.000**	4.8 (9.0)	**[1.2;8.5]**	**0.011**	1.4 (11.2)	[-2.8;5.7]	0.494	**0.022**	3.4 (8.9)	[-1.0;8.2]	0.153	4.9 (11.0)	**[0.7;10.1]**	**0.019**	-1.3 (9.5)	[-6.5;2.9]	0.629
Knee contribution (% overall work)	61.2 (11.2)	70.8 (10.5)	59.5 (9.8)	66.6 (7.7)	64.0 (8.9)	65.8 (9.0)	-9.5 (14.8)	**[-15.3;-3.8]**	**0.002**	-7.1 (12.7)	**[-12.2;-2.0]**	**0.009**	-1.8 (10.9)	[-6.0;2.3]	0.367	0.258	-3.2 (12.6)	[-9.1;3.6]		-3.2 (13.9)	[-10.9;2.0]		1.0 (16.9)	[-4.8;8.2]	
Ankle contribution (% overall work)	13.3 (11.9)	10.8 (8.9)	13.2 (9.2)	10.9 (7.2)	14.1 (9.1)	13.7 (8.6)	2.5 (14.7)	[-3.2;8.2]	0.374	2.3 (10.9)	[-2.1;6.6]	0.299	0.4 (8.5)	[-2.8;3.7]	0.796	0.925	-0.2 (15.1)	[-7.3;5.6]		-1.7 (13.5)	[-7.5;5.6]		0.3 (16.5)	[-6.5;6.7]	
Loading rate (BW/s)	70.3 (24.7)	66.5 (21.0)	72.9 (21.6)	75.4 (21.5)	77.3 (20.6)	81.2 (24.1)	3.8 (21.9)	[-4.7;12.3]	0.370	-2.5 (21.0)	[-10.9;6.0]	0.553	-3.9 (16.3)	[-10.1;2.3]	0.211	0.487	-6.7 (29.1)	[-21.2;7.1]		-3.3 (33.0)	[-18.9;10.0]		-1.5 (31.3)	[-17.2;11.9]	

GRF, ground reaction force; BW, body weight

* valid kinetic data: non imputed valid data available after single-leg hop data collection

° imputed kinetic data: invalid kinetic data (i.e., case where no isolated landing on a force plate but a successful single-leg hop task was performed) was imputed using a linear regression model fitted onto the valid data set (Model input parameters for patients: age, sex, height, weight, days from injury to surgery, month post op, hop distance; Model input parameters for controls: age, sex, height, weight, hop distance)

one-way analysis of variance (ANOVA) with Tukey post hoc tests

Changes in significancy between the analysis of the valid and the imputed kinetic data set are indicated in grey
